# Alterations in microhabitat can impact litter decomposition by modifying the litter C/N ratio and regulating soil microbial activity

**DOI:** 10.3389/fpls.2025.1660144

**Published:** 2026-01-20

**Authors:** Meiqi Li, Jianian Wang, Xiangyi Li

**Affiliations:** 1 Xinjiang Key Laboratory of Desert Plant Roots Ecology and Vegetation Restoration, Xinjiang Institute of Ecology and Geography, Chinese Academy of Sciences, Urumqi, China; 2 State Key Laboratory of Desert and Oasis Ecology, Xinjiang Institute of Ecology and Geography, Chinese Academy of Sciences, Urumqi, China; 3 National Field Scientific Observation and Experimental Station of Cele Desert Grassland Ecosystem, Cele, China; 4 University of Chinese Academy of Sciences, Beijing, China

**Keywords:** litter decomposition, microhabitats, nutrient release, photodegradation, sand burial, soil microorganisms

## Abstract

**Introduction:**

Under extreme drought conditions, the mechanisms of litter decomposition and the associated microbial activities differ significantly from those in non-drought regions. These differences are primarily attributed to reduced precipitation, sparse vegetation cover, intense solar radiation, and unstable soil environments. However, it remains unclear how alterations in litter properties and soil microbial communities—induced by changes in the microenvironment under extreme drought—affect the processes of litter decomposition.

**Methods:**

To address this issue, we selected two distinct habitat types, applied two sand-burial treatments (surface vs. 15 cm depth), and employed the litterbag method to investigate how variations in microenvironmental conditions influence litter decomposition via changes in litter quality and soil microbial communities.

**Results:**

The results indicated that in vegetated areas, sand burial enhanced nitrogen (N) release from litter by 16.56%, accelerated carbon (C) release by 13.13%, and significantly increased mass loss by 7.50%. Structural equation modeling further revealed that *Actinobacteria* and *Ascomycota* significantly promoted litter decomposition in vegetated areas by enhancing lignin degradation. In contrast, the higher decomposition rate of surface litter in non-vegetated areas (litter decomposition rate (*k*): 0.421 vs. 0.275) suggests that abiotic factors are more influential in driving decomposition on exposed sandy land. A 29.37% increase in lignin breakdown, likely due to photodegradation, may be the primary mechanism accelerating surface litter mass loss in these areas.

**Discussion:**

In summary, modifications to the microenvironment influence litter decomposition by altering nutrient release and the composition of soil microbial communities, ultimately affecting ecosystem C and N cycling in arid environments.

## 1 Introduction

Litter decomposition is a critical process for transferring C from terrestrial ecosystems to the atmosphere (Austin and Ballaré, 2024), accounting for approximately 70% of the annual global C flux (Zeng et al., 2024). Given that arid regions cover more than 45% of the Earth’s land surface (Yao et al., 2020), decomposition dynamics in these environments play a substantial role in the global C cycle. In arid ecosystems, litter decomposition is shaped by unique environmental constraints, including limited precipitation, intense solar radiation, and unstable soil structure, that differ significantly from the conditions found in non-arid environments (Wang et al., 2021; Li et al., 2024). However, most research on litter decomposition has concentrated on humid and semi-humid ecosystems (Craig et al., 2022; de Oliveira et al., 2023), while studies in arid ecosystems remain limited. Particularly in the context of changing arid environments, the dynamics and mechanisms of litter decomposition in arid ecosystems remain poorly understood. Thus, investigating the response mechanisms of the litter decomposition process to changes in dryland habitats can enhance our understanding of nutrient cycling within ecosystems and contribute to more accurate future predictions of C dynamics in terrestrial ecosystems.

The rate of litter decomposition is primarily determined by the synergistic interactions among environmental factors, litter quality, and soil microbial communities (Liu et al., 2024). At large spatial scales, environmental factors—including temperature, moisture, and soil pH — are widely recognized as the dominant drivers of litter decomposition (Berg et al., 1993; Sun et al., 2023). Studies have shown that warming can indirectly affect microbial activity and the ecological processes they mediate, such as litter decomposition, by directly modifying soil moisture and temperature, which in turn influences the availability of carbon sources and energy required for microbial growth and metabolism (Rowland et al., 2018; Hakobyan et al., 2023). Additionally, in precipitation-sensitive arid ecosystems, increased precipitation can enhance microbial decomposition while simultaneously suppressing photodegradation (Huang and Li, 2017; Qu et al., 2021; Austin and Ballaré, 2024). Conversely, precipitation may also intensify the leaching of compounds from litter, thereby increasing its susceptibility to photodegradation (Smith et al., 2010; Fan et al., 2021; Li et al., 2022). Soil pH further indirectly influences decomposition rates by affecting the chemical forms of nutrients and their bioavailability (Johnson et al., 2013; Wang et al., 2024). Furthermore, anthropogenic activities, such as nitrogen and acid deposition, impose additional effects on the decomposition process (Li et al., 2022), thereby further complicating it. Despite this, our understanding of how environmental changes in arid regions influence soil microorganisms, and consequently affect litter decomposition, remains limited. This uncertainty hampers our ability to assess the role of diverse microenvironments in regulating decomposition processes and constrains accurate evaluation of C cycling in these ecosystems.

Previous studies on litter decomposition have primarily emphasized the quality of litter and soil microorganisms within specific environmental contexts (Cornwell et al., 2008; Yang et al., 2019a), relatively little is known about how decomposition dynamics vary across different habitat types. Jonsson and David (2008) revealed that in typical meadow ecosystems, favorable environmental conditions, such as higher temperatures, lower humidity, and a reduced C/N ratio, strongly promote litter decomposition. In contrast, litter with a high C/N ratio decomposes more slowly, exhibiting a stronger negative correlation with decomposition rates. In marsh meadows, where temperatures and humidity are lower, decomposition proceeds at a slower rate and the correlation with C/N ratio is weaker. These findings suggest that environmental conditions may exert a stronger influence on decomposition than litter chemical properties such as C/N ratio or lignin content (Berg, 2000). In arid ecosystems, Yang et al. (2019b) showed that nutrient release from litter is closely tied to its structural composition and stoichiometric traits (e.g., total C/N and C/P ratios), and that environmental conditions can modify the release process. Across various ecosystems, studies have shown that litter decomposition rate responses to shifts in litter traits vary considerably depending on ecosystem type and soil characteristics (Alonso et al., 2018; de Oliveira et al., 2023). In arid regions, limitations in soil moisture and nutrients create stark contrasts between vegetated and non-vegetated areas (Wang et al., 2013; Zhao et al., 2013). Additionally, frequent sandstorms in unvegetated landscapes can disperse litter across wide areas, introducing variability into decomposition dynamics. However, the drivers of such variability remain poorly understood. Therefore, comprehensive studies are needed to clarify how litter decomposition in arid regions responds to changing environmental conditions, knowledge that is essential for improving the accuracy of biogeochemical models in dryland ecosystems.

To date, limited research has examined litter decomposition under varying site conditions, with most studies focusing primarily on surface soils. However, variations in soil properties across different depths suggest that soil layers may respond differently to litter decomposition (Jia et al., 2015; He et al., 2023). In extremely dry and arid regions, soil nutrient availability is low, and organic matter content is minimal (Gao et al., 2022; Yuan et al., 2024). Frequent wind activity results in localized sand movement and the burial of litter, making sand burial a common phenomenon in these ecosystems (Wang et al., 2013; Lee et al., 2014). Sand burial introduces complex microenvironmental changes by reducing light intensity and soil temperature while increasing soil moisture (Vleeshouwers, 1997; Teraminami et al., 2013). These environmental shifts can significantly influence litter mass loss (Danger et al., 2012). Some studies have shown that in arid ecosystems, the improved temperature and moisture conditions following sand burial can enhance microbial activity and accelerate the degradation of litter compared to surface-exposed litter (Austin et al., 2016; Luo et al., 2017). However, research in extremely arid conditions has demonstrated that surface litter decomposition can also be rapid (Fan et al., 2021). This may result from “direct photolysis,” whereby intense solar radiation breaks down recalcitrant compounds such as lignin and hemicellulose, facilitating subsequent microbial decomposition (Qu et al., 2021; Austin and Ballaré, 2024). Notably, Li et al. (2007) reported no significant difference in litter mass loss between buried treatments, possibly because these treatments were not exposed to radiation, resulting in low decomposition rates and minimal variation. Thus, how microorganisms in different soils respond to distinct microhabitats, and how these interactions influence litter decomposition, remain poorly understood. Further research is needed to clarify the underlying mechanisms driving these processes.

In this one-year litter decomposition study conducted in an extremely arid ecosystem, we aimed to examine how changes in litter properties and soil microbial communities in response to microenvironmental alterations affect the mechanisms of litter decomposition. We tested the following hypotheses: 1) Under extreme environmental conditions, microenvironmental variation is the primary driver influencing litter mass loss, C/N ratio, and lignin release or sequestration; 2) Soil microorganisms play a crucial role in litter decomposition when buried at a depth of 15 cm in both vegetated and non-vegetated habitats; and 3) On the surface of bare sandy soils, photodegradation-driven lignin breakdown is the dominant mechanism accelerating litter decomposition.

## 2 Materials and methods

### 2.1 Study site

This study was conducted at the National Field Observation and Research Station for the Cele Desert Grassland Ecosystem, located between 35.30°–39.50°N and 80.06°–82.18°E (**Supplementary Figure S1**). The region is located at an elevation of approximately 1300 m, characterized by a typical continental arid climate with extremely limited water availability. The mean annual precipitation is approximately 42.6 mm, while annual evaporation exceeds 2600 mm. Temperature extremes range from -23.9 °C to 41.9 °C. The dominant soil type is sandy soil, characterized by a low organic matter content of approximately 0.8%. The dominant landscape consists of wind-blown sandy formations with sparse vegetation, primarily composed of *Karelinia caspica*, *Alhagi* sp*arsifolia*, *Populus euphratica*, and *Calligonum mongolicum* (Gao et al., 2022; Gao and Zeng, 2023; Li et al., 2024).

### 2.2 Litter collection and experimental design

Leaves from *Alhagi* sp*arsifolia* were selected for litter collection. From early to mid-October 2021, (50 × 50) cm collection baskets were placed beneath the plant canopy to gather freshly senesced litter. After collection, the litter was thoroughly mixed, impurities were removed, and samples were oven-dried at 75 °C for 48 hours. On October 25, 2021, approximately 15 g of dried litter was weighed using an electronic balance accurate to 0.01 g and sealed into (15 × 15) cm nylon mesh bags (1 mm aperture). Three samples were retained to analyze initial litter C, N, C/N ratio, lignin content, and the lignin/N ratio (**Table 1**).

**Table 1:** Initial chemical composition of the litter (*n* = 3).

Different species	C (g/kg)	N (g/kg)	Lignin (g/kg)	Cellulose (g/kg)	Hemicellulose (g/kg)	C/N	Lignin/N
*Alhagi* *sparsifolia*	403.71 ± 3.81	15.53 ± 0.33	15.28 ± 3.67	54.75 ± 2.9	30.07 ± 1.52	26.02 ± 0.46	9.84 ± 2.9

The experimental site was located in a transitional zone between oasis and desert environments. Two distinct habitat types were established: a non-vegetated area and a vegetated area dominated by *Alhagi* sp*arsifolia*. On October 31, 2021, litterbags were deployed in both habitats at two depths, on the soil surface and buried at 15 cm, to simulate natural decomposition processes. The bags were secured with nails to prevent displacement. Litter samples were collected six times from each habitat, with five replicates per treatment. Each sampling quadrat measured 50 × 50 cm and was spaced 1 m apart, resulting in a total of 120 litter bags used in the experiment.

### 2.3 Litter and soil sample collection

Samples were collected during the 5th, 7th, 8th, 10th, 11th, and 12th months of the decomposition process. A total of 20 litter decomposition mesh bags were sampled each month. The study measured the contents of C, N, lignin, and the ratios of C/N and lignin/N after decomposition. Additionally, litter mass loss and decomposition rates were evaluated. Total carbon (TC) and total nitrogen (TN) in both litter and soil were determined using a C and N elemental analyzer (Elementar, Hanau, Germany). Lignin content was analyzed with a cellulose analyzer (FOSS, Hilleroed, Denmark).

In December, soil samples were collected from litterbag surfaces with gloves worn throughout the process. The microbial DNA was extracted from soil samples collected adjacent to the decomposition bags, specifically from the peripheral zone located 1–2 cm from the bag edges, excluding material from within the bags themselves. To ensure spatial independence among biological replicates, sampling points were separated by at least 50 cm to minimize potential cross-contamination via diffusion of water, nutrients, or microorganisms. Each replicate was associated with an independently deployed litter bag and its surrounding soil matrix. Sampling order was randomized to avoid systematic procedural bias. Within each sampling unit, subsamples were collected uniformly around the bag (e.g., at cardinal directions) and combined into a single composite sample to enhance representativeness and reduce local variability. Each time, 20 litterbags were retrieved from the plots and immediately placed in iceboxes for transport to the laboratory. Surface soil was carefully brushed off each bag using a clean brush and transferred into 1.5 mL centrifuge tubes. Three replicates per treatment were labeled and stored at −20 °C. All samples were transported to the sequencing facility within one week using dry ice.

DNA was extracted using the OMEGA Soil DNA Kit (D5635-02, Omega Bio-Tek, Norcross, GA, USA). Initial quantification and assessment of DNA purity were performed using NanoDrop spectrophotometry, followed by precise concentration measurement with Qubit fluorometry. Samples were required to meet the following quality criteria: an OD260/280 ratio between 1.8 and 2.0, an OD260/230 ratio greater than 1.8, and a DNA concentration of at least 10 ng/μL. Those failing to meet these thresholds were either re-extracted or concentrated to ensure sufficient quality and quantity for downstream applications. The bacterial 16S rRNA gene V3-V4 region was amplified using primers 338F (5’-barcode+ACTCCTACGGGAGGCAGCA-3’) and 806R (5’-GGACTACHVGGGTWTCTAAT-3’), yielding an amplicon of approximately 468 bp. Amplification of the fungal ITS1 region was conducted with primers ITS5 (GGAAGTAAAAGTCGTAACAAGG) and ITS2 (GCTGCGTTCTTCATCGATGC), producing fragments typically ranging from 300 to 400 bp in length. The PCR reaction was carried out in a 25 μL system containing template DNA, primers, dNTPs, high-fidelity DNA polymerase, and corresponding buffer. The thermal cycling conditions consisted of an initial denaturation at 95 °C for 5 min, followed by 30 cycles of denaturation at 95 °C for 30 s, annealing at 55 °C for 30 s, and extension at 72 °C for 45 s, with a final extension at 72 °C for 5 min, and then held at 12 °C. To monitor potential contamination, a no-template negative control (NTC) was included in each PCR batch, in which sterile water replaced the DNA template to detect exogenous contamination from reagents or procedural handling. Furthermore, in selected batches, a Mock Community—comprising a defined mixture of genomic DNA from multiple standard bacterial strains at known ratios—was amplified alongside samples to assess the accuracy and potential bias of the sequencing and bioinformatics pipeline. All PCR products were verified by agarose gel electrophoresis to confirm amplification specificity and fragment size, then purified using magnetic beads, and pooled in equimolar amounts for library construction. Sequencing was performed on the Illumina MiSeq or NovaSeq platform using paired-end chemistry with read lengths of 2 × 250 bp or 2 × 300 bp. For diversity analysis, rarefaction was applied to all samples at a depth of 10,000 high-quality sequences per sample to ensure comparability of α- and β-diversity metrics. Operational taxonomic units (OTUs) were clustered at a 97% sequence similarity threshold using high-quality sequences. Specifically, pairwise distances were calculated using USEARCH or VSEARCH algorithms, and sequences with ≥97% similarity were grouped into the same OTU via UPGMA or hierarchical clustering. An OTU table was subsequently generated to summarize the abundance distribution of microbial taxa across all samples.

### 2.4 Statistical analysis

Data were processed using Excel 2019, and all statistical analyses and graphical visualizations were performed using R software (version 4.1.0). The decomposition rate constant (*k*) was calculated using a single exponential decay model (Olson, 1963): $X_{t}=X_{0}\;e^{-kt}$. The mass remaining (*MR*) was calculated as the ratio of residual litter mass to initial litter mass after a given duration of decomposition (Gong et al., 2023): $MR=(X_{t}/X_{0})\times 100\%$, where *X*_0_ (g) is the dry weight of litter at the beginning of the experiment, *X_t_* (g) is the residual mass of litter at decomposition time *t*, e is the base of the natural logarithm, and *k* is the litter decomposition rate. The release or accumulation of each element in the litter can be quantitatively characterized using the nutrient accumulation index (*R*). An *R* < 100% indicates net nutrient release from the litter, while an *R* > 100% indicates net nutrient accumulation (McGroddy et al., 2004): $R=M_{t}\times C_{t}/(M_{0}\times C_{0})$ × 100%, where *C*_0_ (mg.g^-1^) and *M*_0_ (g) denote the initial elemental concentration and dry mass of the litter, respectively, whereas *C_t_* (mg.g^-1^) and *M_t_* (g) represent the corresponding concentration and dry mass at time t.

One-way ANOVA was conducted to assess the effects of different treatments on litter mass remaining, element remaining, decomposition rate, abundance of dominant microorganisms, and α-diversity, followed by Tukey’s *post-hoc* test for pairwise comparisons using the “*multcomp*” package in R. A linear mixed-effects model (LMM) was employed to assess the effects of habitat type (H, vegetated vs. non-vegetated areas), burial depth (D, surface vs. 15 cm depth), sampling time (T, months 5, 7, 8, 10, 11, and 12), and their interactions on litter mass loss, decomposition rate constant (*k*), nutrient retention, abundance of dominant microbial taxa, and alpha diversity. Fixed effects were included to quantify both the individual and interactive influences of these factors on the response variables. Specifically, two-way interactions (habitat type × burial depth, habitat type × sampling time, burial depth × sampling time) and the three-way interaction (habitat type × burial depth × sampling time) were incorporated to examine potential synergistic or context-dependent effects. To account for inherent variability among habitat units, habitat type was modeled as a random intercept. The models were fitted using the lmer function in the “*lmerTest*” package in R, with parameter estimation based on restricted maximum likelihood (REML). Model selection was guided by Akaike’s Information Criterion (AIC) and Bayesian Information Criterion (BIC), favoring the most parsimonious model with optimal fit. Comprehensive diagnostic checks were conducted to verify model assumptions, including assessments of residual normality, homogeneity of variance, and the distribution of random effects. For statistically significant main effects or interactions, *post-hoc* pairwise comparisons were performed using Tukey’s Honestly Significant Difference (HSD) test, implemented via the emmeans package in R to control for family-wise error rates. Additionally, linear regression analysis was performed to assess the relationships between litter traits and residual mass.

To explore microbial community overlap, the *VennDiagram* package in R was used based on OTU abundance data across all samples, with each group treated as a distinct set. OTU counts and shared membership among sets were calculated accordingly. The OTU table was further processed in R to assess microbial heterogeneity across treatments and to construct a relative abundance table at the phylum level. α-diversity metrics, including Chao1, Observed_species, Shannon, and Simpson indices, were calculated at the OTU table using QIIME2. Visualization of α-diversity and taxonomic composition was conducted using the *ggplot2* package in R, presented as bar charts and boxplots. To evaluate the patterns of community β-diversity variation and its driving factors, the Bray-Curtis dissimilarity matrix was calculated to quantify compositional differences among species or functional groups and visualized using non-metric multidimensional scaling (NMDS). The statistical significance of group-level differences was assessed through permutational multivariate analysis of variance (PERMANOVA). Detailed methodological procedures are provided in the **Supplementary Materials**. Relationships between microbial α-diversity, soil physicochemical properties, and litter mass loss were assessed using Spearman’s correlation. Pearson correlation coefficients were also calculated to determine associations among litter mass loss, litter characteristics, and microbial community variables. Finally, structural equation modeling (SEM) was conducted to evaluate the direct and indirect effects of different microhabitat on litter mass loss and soil nutrient cycling through litter characteristics and microbial abundance. Based on prior knowledge and literature, a hypothesized model was constructed, treating litter characteristics and microbial abundance as latent variables (**Supplementary Figure S2**). The SEM model was evaluated using AMOS 22.0 software (SPSS, IBM, NY, USA).

## 3 Results and analysis

### 3.1 Characteristics of leaf decomposition across habitats

**Figure 1:**
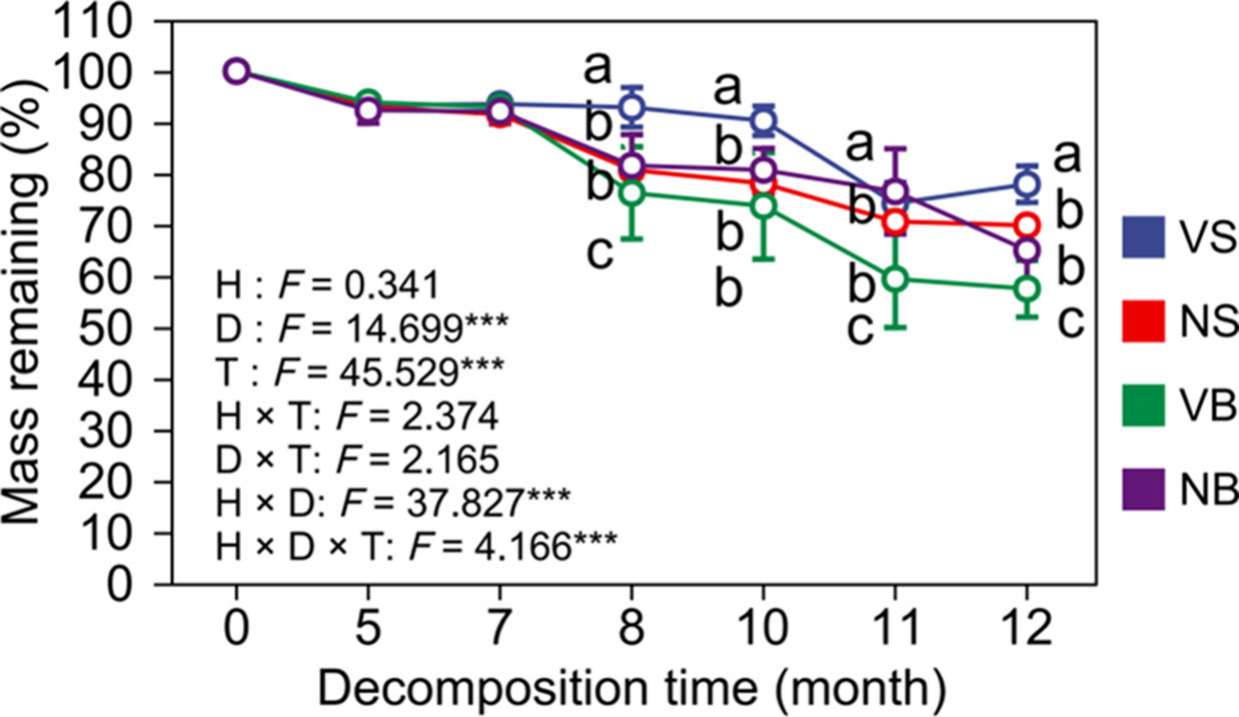
Temporal dynamics of litter mass under different treatments. Values are means and standard errors (*n* = 3, *P* < 0.05). V = vegetation, N = non-vegetated, S = surface, and B = 15 cm burial depth.

Throughout the decomposition period, litter mass consistently declined over time (**Figure 1**). Habitat type did not significantly influence litter mass remaining (*P* > 0.05). However, the interaction between habitat type and burial depth had a significant effect on litter mass dynamics, with the magnitude of this interactive effect increasing over time (*P* < 0.05). By December, surface litter in the non-vegetated area decomposed more rapidly than in the vegetated area, with a higher residual mass observed in the vegetated habitat (mass remaining: 79.55 ± 14.32% > 70.08 ± 8.11%). In contrast, at a burial depth of 15 cm, litter in the vegetated area exhibited greater mass loss, with a lower residual mass compared to the non-vegetated area (mass remaining: 57.29 ± 4.56% < 68.52 ± 6.71%) (*P* < 0.05; **Table 2**).

**Table 2:** Decomposition rate of litter under different habitats.

Treaments	Surface		Sand burial	
	*k* (g.g^-1^.a^-1^)	*r* ^2^	*k* (g.g^-1^.a^-1^)	*r* ^2^
Vegetation area	0.275± 0.03b	0.629	0.637± 0.02a	0.695
Non-vegetation area	0.421± 0.02a	0.807	0.415± 0.02b	0.766

Different letters indicate significant differences among different habitats. Values are means and standard errors (*n* = 3, *P* < 0.05).

### 3.2 Response of residual nutrients to different habitats

**Figure 2:**
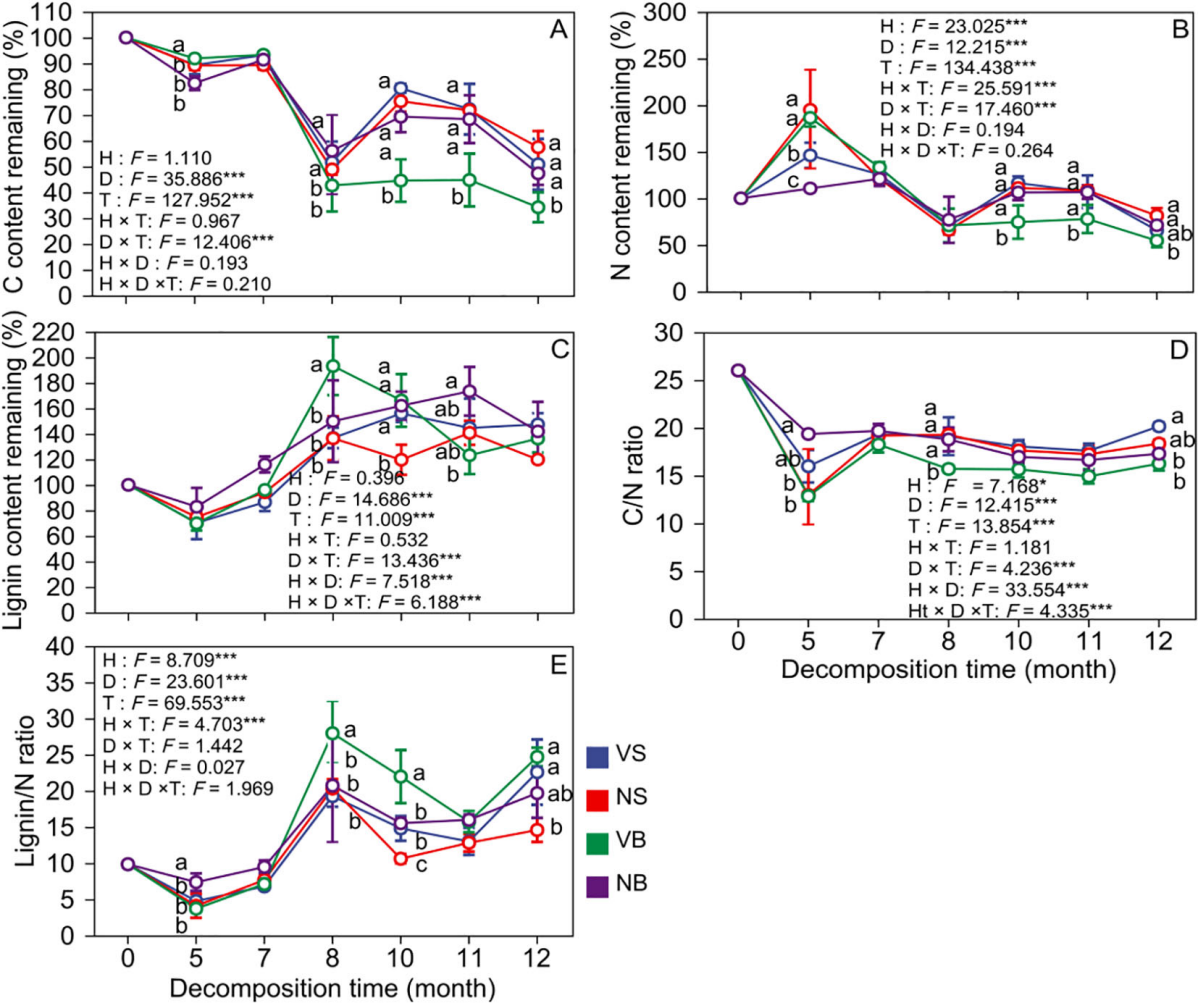
Dynamic changes of litter C **(A)**, N **(B)**, lignin **(C)**, C/N ratio **(D)**, and lignin/N ratio **(E)** under different habitats and burial depths. Different letters indicate significant differences among different treatments. Values are means and standard errors (*n* = 3, *P* < 0.05). V = vegetation, N = non-vegetated, S = surface, and B = 15 cm burial depth.

**Figure 3:**
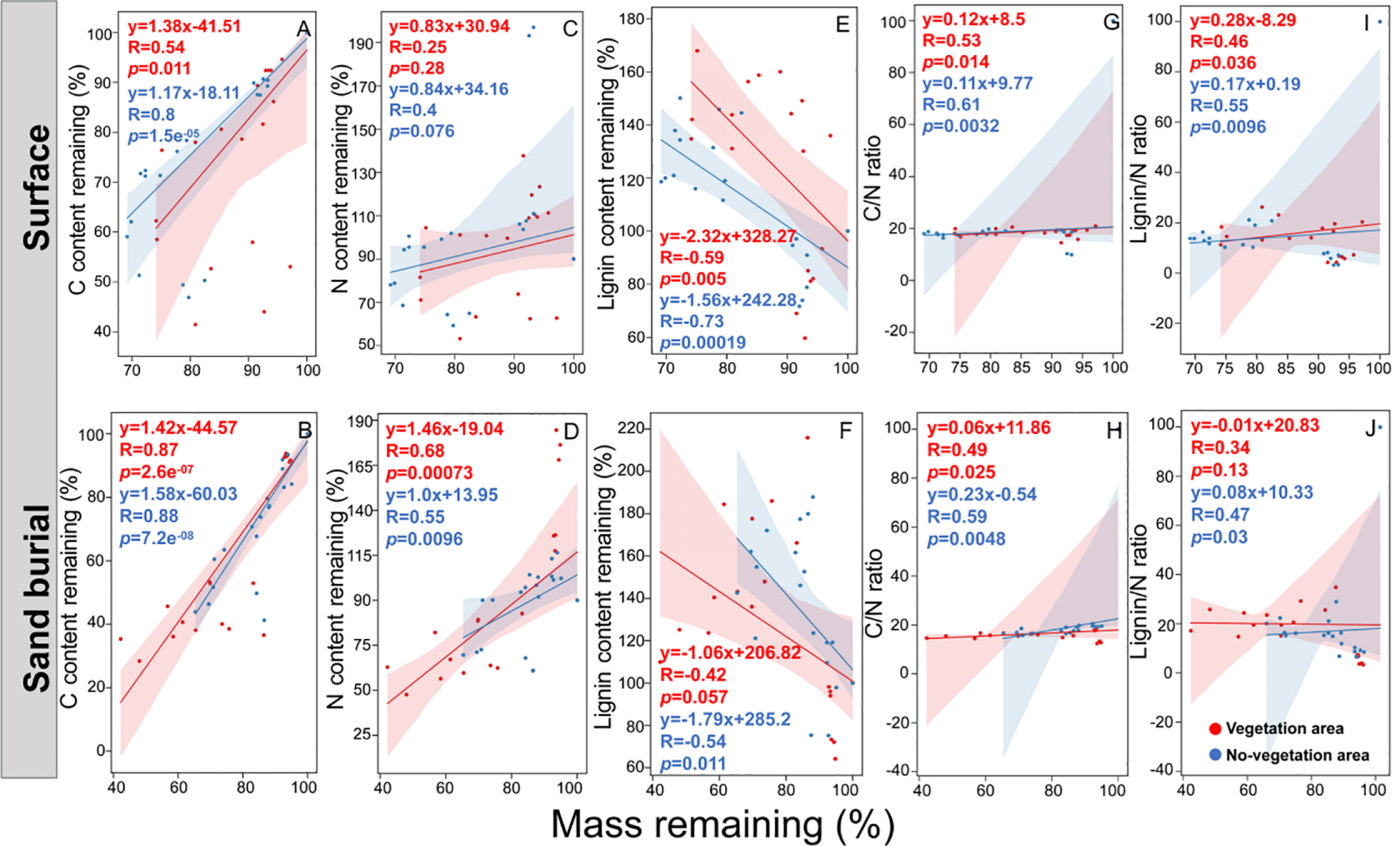
Correlations between litter C content remaining **(A, B)**, litter N content remaining **(C, D)**, litter C/N ratio **(E, F)**, litter lignin content remaining **(G, H)**, and litter lignin/N ratio **(I, J)** with litter mass remaining.

During litter decomposition, C content exhibited a temporal trend similar to that of mass loss across all treatments (**Figure 2A**, **3A, B**). After 12 months, the average remaining C content was 54.17 ± 5.42% and 40.76 ± 4.12%, respectively. The N residue showed an initial increase followed by a decline under both burial depths. Habitat has a highly significant influence on N residue (*P* < 0.001; **Figures 2B**, **3C, D**). After 12 months, N residue in the non-vegetated area was significantly 15.81% and 16.56% higher than in the vegetated area for surface and buried litter, respectively (*P* < 0.05; **Figure 2B**). Habitat also significantly affected the litter C/N ratio (*P* = 0.024; **Figure 2D**). The C/N ratio decreased over time under both burial depths. After 12 months, surface litter in the non-vegetated area had a C/N ratio 1.81 units lower than in the vegetated area, whereas the trend reversed at the 15 cm depth, though the difference was not significant (*P* > 0.05; **Figure 2D**). Additionally, the C/N ratio was significantly negatively correlated with litter mass (**Figure 3G, H**), suggesting that sand burial facilitated C/N ratio reduction, thereby promoting mass loss. Lignin content exhibited a continuous accumulation trend across both burial depths. Habitat did not significantly influence lignin residue (*P* = 0.054; **Figure 2C**). However, after 12 months, surface litter in the non-vegetated area had significantly 29.37% less lignin residue than in the vegetated area (*P* < 0.05), though this difference was not significant at the 15 cm burial depth (*P* > 0.05; **Figure 2C**). The lignin/N ratio initially declined and subsequently increased during decomposition. Habitat significantly affected the lignin/N ratios (*P* < 0.001; **Figure 2E**). After 12 months, litter in the non-vegetated area exhibited lignin/N values 7.99 and 5.00 units significantly lower than those in the vegetated area under surface and buried conditions, respectively (*P* < 0.05; **Figure 2E**). Except for a few exceptions, the lignin content was significantly negatively correlated with litter mass remaining (**Figures 3E, F**). In contrast, the lignin/N ratio was significantly positively correlated with litter mass remaining (**Figure 3I, J**). This pattern may reflect differences in litter chemical quality induced by microhabitat variation.

### 3.3 Response of soil microbial communities to different habitats

**Figure 4:**
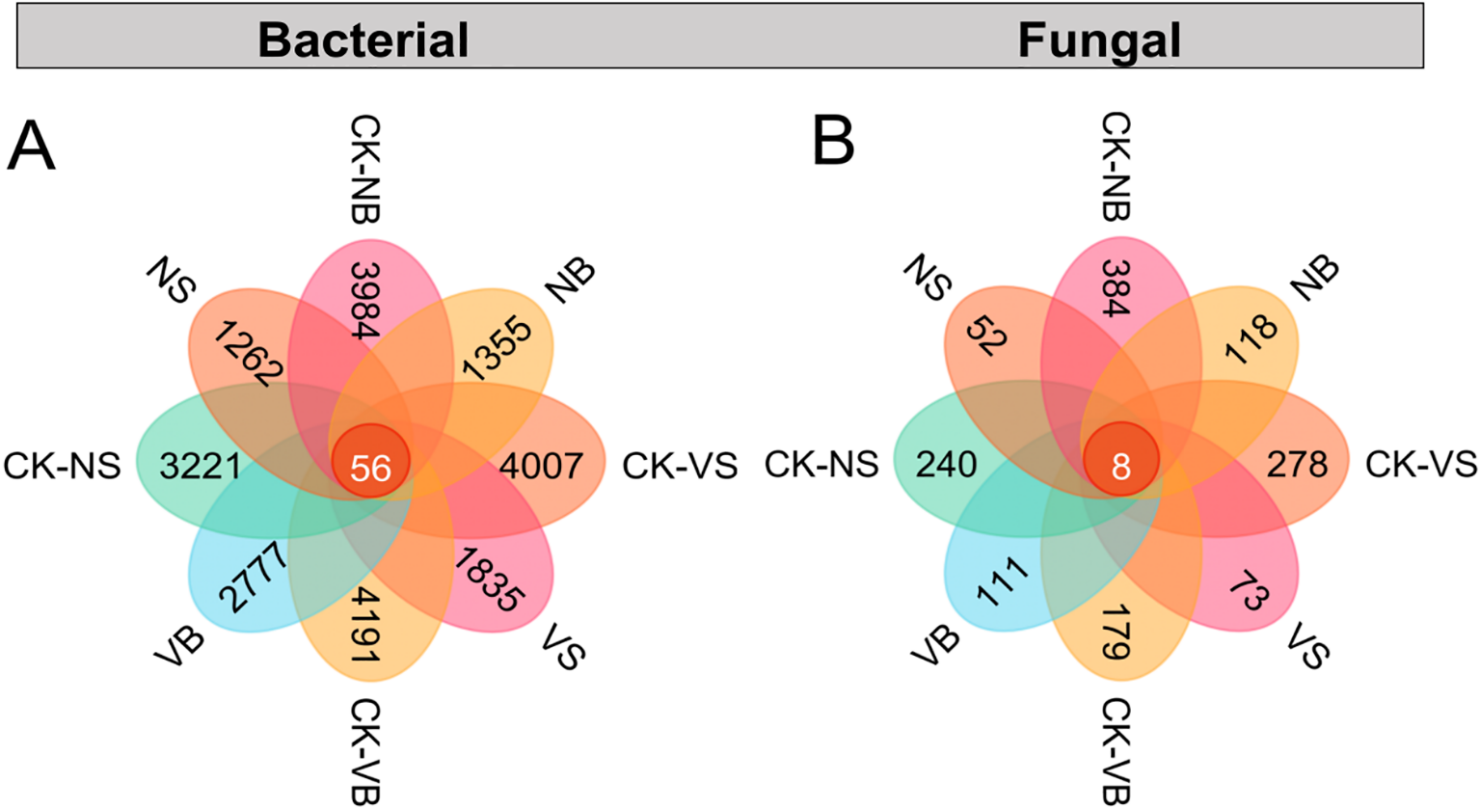
The distribution of shared and unique Operational Taxonomic Units (OTUs) in soil bacterial **(A)** and fungal **(B)** communities. CK = Initial decomposition time. V = vegetation, N = non-vegetated, S = surface, and B = 15 cm burial depth.

**Figure 5:**
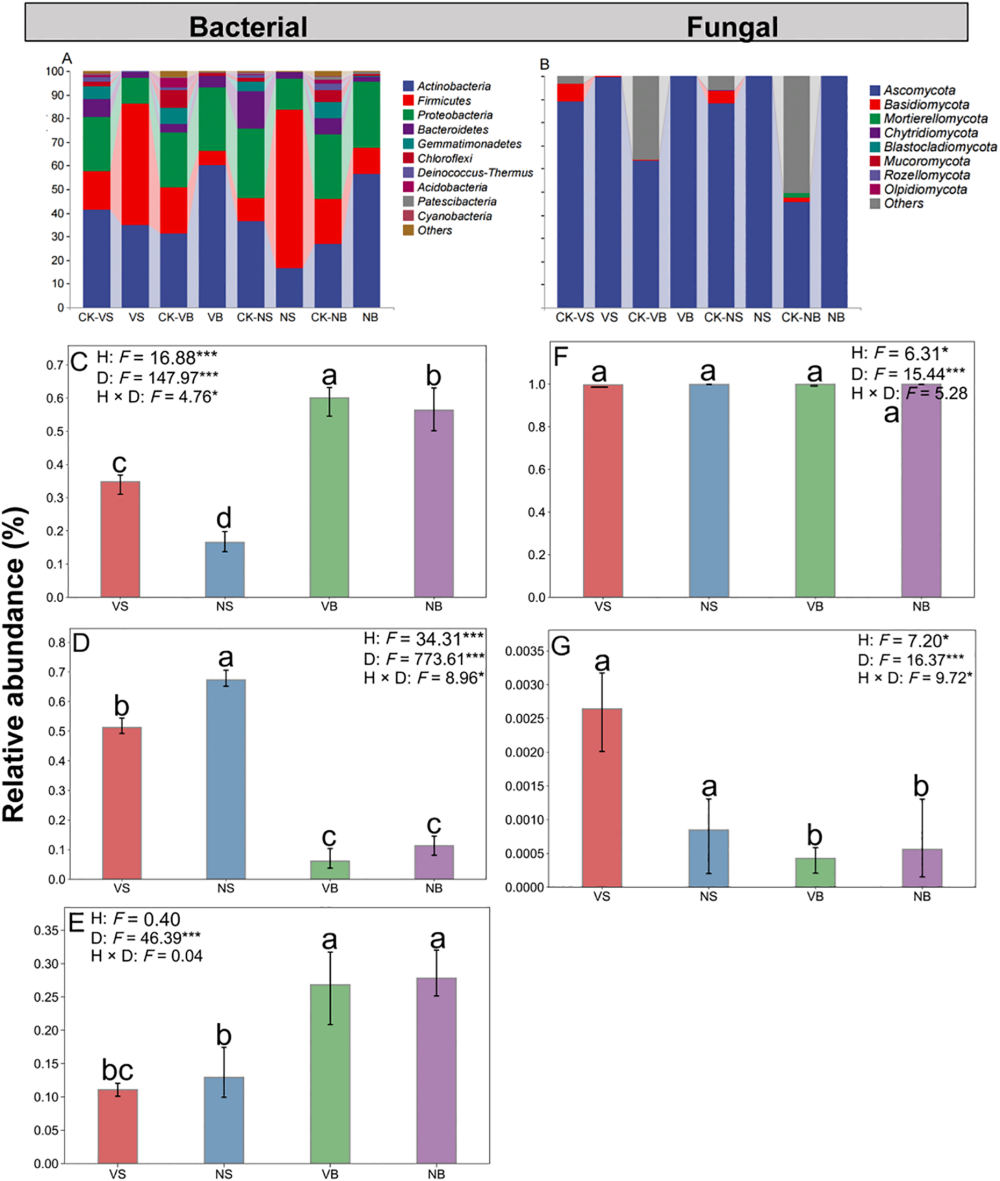
Relative abundance of soil bacterial **(A)** and fungal **(B)** communities across treatments. **(C–E)** show the relative abundances of *Actinobacteria*, *Firmicutes*, and *Proteobacteria*, respectively; panels **(F–G)** display the relative abundances of *Ascomycota* and *Basidiomycota*, respectively. Values are means and standard errors (*n* = 3, *P* < 0.05). CK = Initial decomposition time. V = vegetation, N = non-vegetated, S = surface, and B = 15 cm burial depth.

As illustrated in the petal diagrams, the number of bacterial and fungal OTUs was generally higher in the vegetated area than in the non-vegetated area across both burial depths, with few exceptions (**Figures 4A, B**). We analyzed microbial relative abundance and diversity after 12 months of decomposition, a period characterized by significant changes in soil microbial community structure. The dominant bacterial phyla in the soil during litter decomposition were *Firmicutes*, *Actinobacteria*, and *Proteobacteria* (**Figure 5A**). The habitat significantly influences the relative abundances of *Firmicutes* and *Actinobacteria* (*P* < 0.05; **Figures 5C, D**), but has no significant effect on *Proteobacteria* (*P* > 0.05; **Figure 5E**). At both the surface and 15 cm burial depth, the relative abundance of *Actinobacteria* in vegetated areas was significantly higher than in non-vegetated areas by 18% and 4%, respectively (*P* < 0.05), whereas *Proteobacteria* and *Firmicutes* showed an opposite trend, although these differences were not statistically significant (*P* > 0.05; **Figures 5C-E**). Under the 15 cm sand burial treatment, the relative abundance of *Actinobacteria* increased significantly by 25% in vegetated areas and by 38% in non-vegetated areas, while *Proteobacteria* increased significantly by 16% and 15%, respectively. In contrast, the relative abundance of *Firmicutes* decreased markedly, by 45% and 56% in vegetated and non-vegetated areas, respectively (*P* < 0.05; **Figures 5C-E**). For soil fungi, the dominant phyla were *Ascomycota* and *Basidiomycota* (**Figure 5B**). No significant differences in the relative abundances of *Ascomycota* and *Basidiomycota* were observed between habitats under either burial depth treatment (*P* > 0.05; **Figures 5F, G**). Furthermore, 15 cm sand burial reduced the relative abundance of *Basidiomycota* by 2.21% and 2.87% in vegetated and non-vegetated areas, respectively, while the relative abundance of *Ascomycota* remained largely unchanged and was not significantly affected (*P* > 0.05; **Figures 5F, G**).

**Figure 6:**
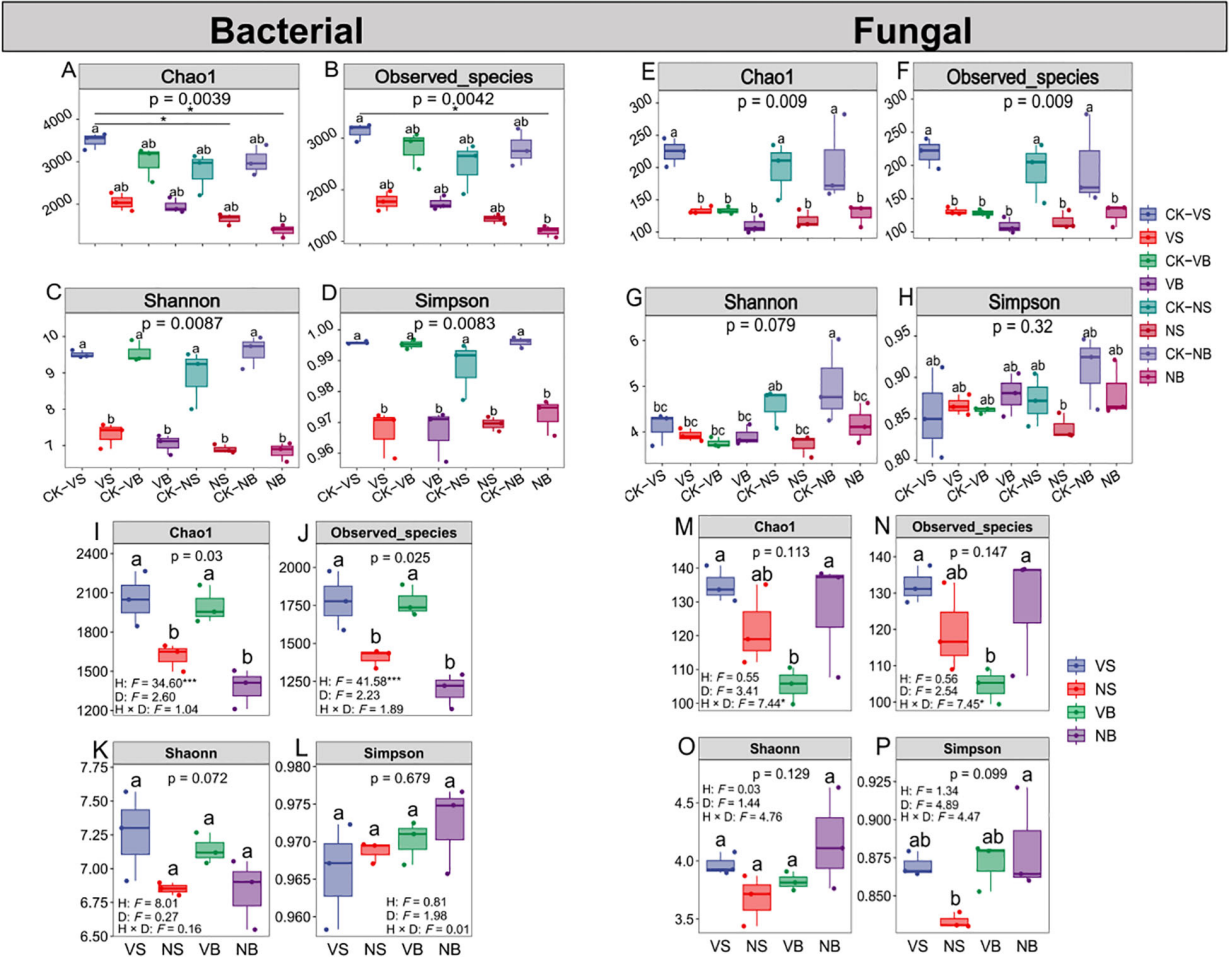
Alpha diversity indices of soil bacterial **(A–D, I–L)** and fungal **(E–H, M–P)** communities across treatments. Values are means and standard errors (*n* = 3, *P* < 0.05). CK = Initial decomposition time. V = vegetation, N = non-vegetated, S = surface, and B = 15 cm burial depth.

The results indicated that, with few exceptions, the α-diversity indices of both bacterial and fungal communities were significantly higher in the vegetated area compared to the non-vegetated area (*P* < 0.05; **Figure 6**; **Supplementary Table S1**). For bacteria, all α-diversity indices were significantly higher in the vegetated area at the surface. However, sand burial at 15 cm depth significantly reduced bacterial α-diversity in both habitats (*P* < 0.05; **Figures 6A–D**). Notably, only the bacterial Chao1 and Observed_species indices exhibited significant differences, with habitat having a significant effect on these α-diversity indices(*P* < 0.05; **Figures 6I–J**). For fungi, α-diversity indices at the surface were consistently higher in the vegetated area than in the non-vegetated area, whereas the opposite trend was observed at 15 cm burial depth, although these differences were not statistically significant (*P* > 0.05; **Figures 6M–P**). To further explore variation in microbial community structure across treatments, we conducted beta diversity analysis based on Bray–Curtis distance matrices. The NMDS revealed clear separation of microbial communities along the NMDS1 and NMDS2 axes (**Supplementary Figure S3**), with all stress values below 0.2, confirming the reliability of the ordination. To statistically validate the observed structural differences, PERMANOVA was performed, revealing significant variation in both bacterial and fungal community compositions across habitat and sand burial treatments (*P* < 0.05). Furthermore, homogeneity of multivariate dispersions was evaluated using the betadisper test, which showed no significant differences in within-group variability among treatments (*P* > 0.05), thereby supporting the robustness of the PERMANOVA results.

### 3.4 Correlation analysis of litter properties, soil chemical components, and microbial communities

**Figure 7:**
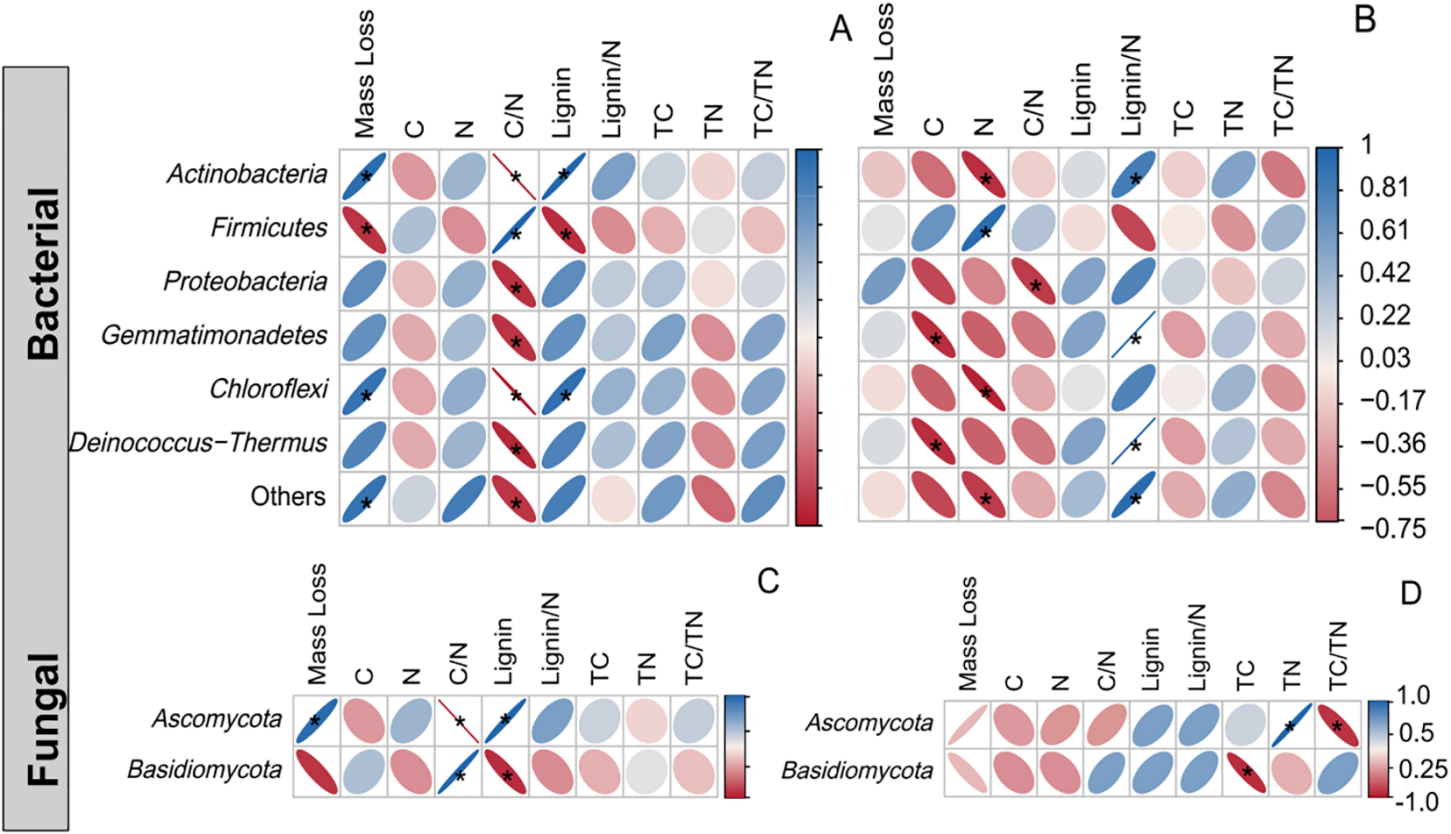
Correlations between the microbial abundance in the vegetated **(A, B)** and non-vegetated areas **(C, D)** and litter mass, litter properties and soil physicochemical variables. Blue represents positive correlations, reds represent negative correlations. Mass loss: litter loss mass; C: litter C concentration; N: litter N concentration; C/N: litter carbon-nitrogen ratio; Lignin: lignin concentration; Lignin/N: lignin-nitrogen ratio; TC: soil total carbon; TN: soil total nitrogen; TC/TN: soil carbon-nitrogen ratio. Significance levels are represented as * *P <* 0.05, ** *P <* 0.01.

The linear mixed model analysis revealed that habitat significantly influenced all dominant microbial groups except *Proteobacteria*, whereas sand burial treatment exerted significant effects on all dominant microorganisms (*P* < 0.05; **Figure 5**). Except for *Firmicutes*, the remaining microbial groups exhibited a significant negative correlation with litter C/N ratio in both habitats (*P* < 0.05), and significant positive correlations with lignin content and lignin/N ratio (*P* < 0.05; **Figure 7**). In the vegetated area, litter mass loss was significantly and positively correlated with *Actinobacteria* and negatively correlated with *Firmicutes* (*P* < 0.05). In contrast, no significant relationships were observed between microbial taxa and litter mass loss in the non-vegetated area (*P* > 0.05). Additionally, *Ascomycota* was significantly positively correlated with both litter mass loss and lignin content, but only in the vegetated area. Notably, microbial α-diversity indices showed no significant correlations with any of the measured physicochemical parameters (**Supplementary Figure S4**), suggesting that variation in microbial diversity alone may not sufficiently influence litter decomposition processes in this system.

**Figure 8:**
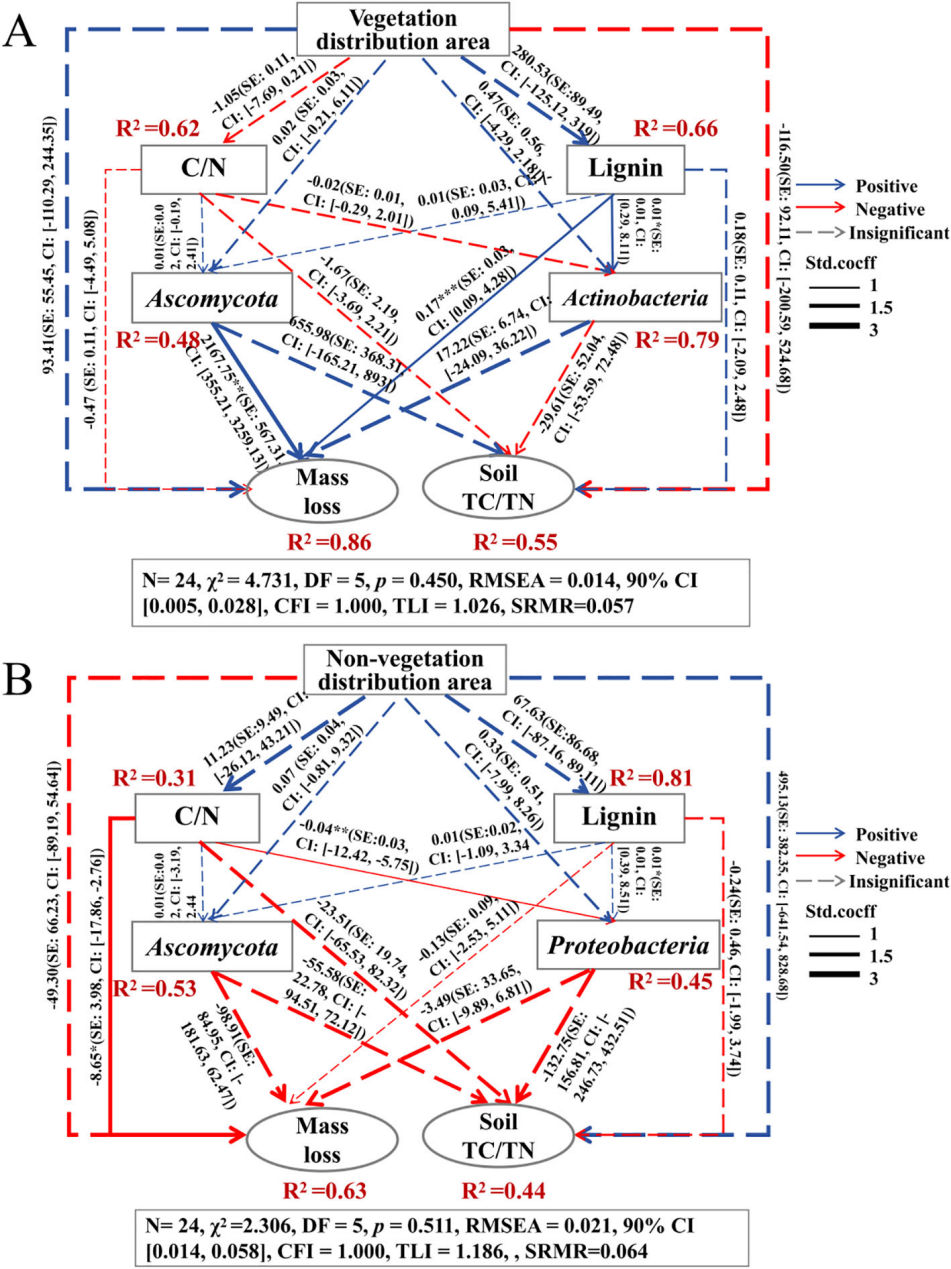
Structural equation model (SEM) demonstrates the influence of vegetation coverage **(A)** and non-vegetation coverage **(B)** on litter chemical composition, soil microbial communities, litter mass loss, and soil nutrient dynamics. Latent variables are represented by ellipses and observed variables by rectangles. Arrow values represent standardized path coefficients, with arrow thickness indicating the magnitude of the coefficients. Blue and red arrows indicate positive and negative correlations, respectively, while solid and dashed arrows denote significant and non-significant relationships. The standardized path coefficients for each path, along with their corresponding standard errors and 95% confidence intervals, are displayed adjacent to the arrows. The R2 below the response variable represents the proportion of variation explained by other correlated variables. C/N: litter carbon-nitrogen ratio; Lignin: lignin concentration; *Ascomycota*: the abundance of the *Ascomycota* phylum; *Actinobacteria*: the abundance of the *Actinobacteria* phylum; *Proteobacteria*: the abundance of the *Proteobacteria* phylum; Mass loss: litter loss mass; Soil TC/TN: soil carbon-nitrogen ratio.

The structural equation model linking litter mass loss and soil nutrients to litter properties, soil microbial abundance, and microenvironmental conditions exhibited a good fit, explaining 86% and 63% of the variation in litter mass loss and 55% and 44% of the variation in soil nutrients, respectively (**Figure 8**). Microhabitat changes primarily regulate litter mass loss and soil TC/TN through indirect pathways by altering the C/N ratio, lignin content, and the relative abundances of *Ascomycota*, *Actinobacteria*, and *Proteobacteria* in the litter (**Figure 8**). Vegetation cover exerts a direct positive effect on litter mass loss and a negative effect on soil TC/TN (*P* > 0.05; **Figure 8A**). It negatively influences the litter C/N ratio while positively affecting lignin content (*P* > 0.05); in turn, a lower litter C/N ratio is negatively associated with litter mass loss, whereas lignin has a positive effect on litter mass loss (*P* > 0.05; **Figure 8A**). Furthermore, vegetation cover enhances the relative abundances of *Ascomycota* and *Actinobacteria* (*P* > 0.05), both of which show positive associations with litter mass loss, with *Ascomycota* showing a significant positive effect (*P* < 0.05) and *Actinobacteria* exhibiting a similar but non-significant trend (*P* > 0.05; **Figure 8A**). In contrast, bare sand has no significant effects on soil microbial communities or litter mass loss (*P* > 0.05), except for a significant negative effect of the C/N ratio on mass loss (*P* < 0.05; **Figure 8B**). Notably, bare sand exerts a direct positive effect on soil TC/TN (*P* > 0.05; **Figure 8B**). Overall, these results suggest that the effects of microhabitat variation on litter decomposition are primarily mediated through changes in litter chemical properties and shifts in the soil microbial community.

## 4 Discussion

### 4.1 Decomposition dynamics of litter in various habitats

Litter decomposition involves dynamic changes in nutrient content, with nutrient release or accumulation primarily governed by the interplay between the chemical composition of the litter and microbial nutrient demands (Dong et al., 2021). In this study, C was consistently released across all treatments, following a pattern similar to that of mass loss (**Figure 2A**, **3A, E**). This phenomenon may be attributed to the high proportion of C-based compounds in the litter (Gong et al., 2020). In contrast to previous studies reporting N enrichment during decomposition (Fan et al., 2021; Yuan et al., 2024), our findings showed net N release under both burial treatments (**Figure 2B**). This deviation may be explained by limited microbial activity in sandy soils under extreme drought conditions, which restricts N immobilization (Fan et al., 2021; Qu et al., 2021). Additionally, the N residue in vegetated areas was significantly lower than in non-vegetated areas, decreasing by 15.81% and 16.56%, respectively. This difference likely reflects the more favorable water and thermal conditions provided by vegetation cover, which enhance microbial activity, accelerate organic matter decomposition, and promote nutrient turnover, thereby increasing N release (Witoon et al., 2016; Fan et al., 2021). As both C and N were released concurrently during decomposition, the litter C/N ratio decreased markedly by the end of the observation period and subsequently stabilized.

Lignin is a complex aromatic polymer that is widely recognized for its high resistance to degradation (de Oliveira et al., 2023). In this study, both lignin residue and the lignin/N ratio exhibited a consistent increasing trend across all treatments (**Figures 2C, E**). This pattern aligns with the findings of Lin and King (2014), who observed similar trends in dry grasslands. One possible explanation is the formation of stable complexes between inorganic N ions in the soil and lignin or its degradation intermediates, which can slow down lignin breakdown (Ye et al., 2022). Notably, lignin residues on the surface of non-vegetated areas were significantly lower than those in vegetated areas, while decomposition rates were markedly higher. In non-vegetated areas, litter is directly exposed to solar radiation, which strongly enhances the photochemical oxidation of lignin and promotes its abiotic degradation, thereby accelerating overall decomposition (Li et al., 2024). A growing body of evidence demonstrates that in arid and semi-arid regions, photolysis plays a significant role in litter mass loss (Day et al., 2019; Austin and Ballaré, 2024). In contrast, vegetation cover reduces the exposure of surface litter to solar radiation (Kan et al., 2015; Zhao et al., 2020), creating a shading effect that attenuates light intensity—particularly in the ultraviolet spectrum—and thereby suppresses photochemical reactions (Qu et al., 2021). Moreover, vegetated areas typically exhibit lower light intensity, higher soil moisture, and more stable temperature regimes—environmental conditions that may collectively slow down both physical weathering and enzymatic degradation of lignin (Lee et al., 2014). Nevertheless, *in-situ* continuous monitoring of ultraviolet radiation remains limited, hindering the establishment of a direct causal link between UV exposure and lignin degradation. Despite this limitation, the observed higher decomposition rates and reduced lignin residues in non-vegetated areas align closely with the expected effects of photolysis, supporting the role of photochemical processes as a key abiotic driver of lignin turnover. Future studies could employ radiation shielding experiments or band-selective filter membranes to quantitatively assess lignin degradation pathways under varying light conditions and elucidate their interactions with biological processes.

### 4.2 Soil microbial communities in response to various habitats

Microbial community analysis did not incorporate direct samples from within the decomposition bags, limiting the ability to accurately evaluate how the internal microenvironment influences the composition of adjacent soil microbial communities. Furthermore, potential micro-scale ecological disturbances induced by the bag material itself could not be excluded. This constraint may weaken the interpretation of material exchange dynamics and microbial migration patterns. Future research should aim to strengthen inferential robustness by concurrently collecting samples from both the litter within the bags and the surrounding soil, enabling paired comparisons that improve the validity and reliability of conclusions.

*Firmicutes*, *Actinobacteria*, and *Proteobacteria* are the predominant bacterial phyla in the soils of many desert vegetation ecosystems (García-Palacios et al., 2016). This study further confirmed that these phyla dominated under all treatments. Across both burial depths, the abundance of *Firmicutes* and *Proteobacteria* was lower in vegetated areas compared to non-vegetated areas (5.21–16.1% and 1.85–0.46%, respectively), whereas *Actinobacteria* showed the opposite trend, with a higher abundance in vegetated areas (3.69–18.27%) (**Figures 5C-E**). These patterns may indicate partial niche overlap or competitive interactions between *Firmicutes* and *Actinobacteria* (or *Proteobacteria*), likely due to similarities in resource acquisition and utilization strategies (Wang et al., 2015). Additionally, microbial groups differ in their nutrient requirements and adaptability to environmental conditions (Dong et al., 2021, 2024), which may account for their varied responses to habitat changes (Guan et al., 2022; Liu et al., 2024). In the fungal community, *Ascomycota* and *Basidiomycota* were the dominant phyla (**Figure 5B**), consistent with previous findings by Zheng et al. (2021). Correlation analysis revealed that *Ascomycota* was positively associated with lignin content in both habitats. Both *Ascomycota* and *Basidiomycota* are known for their strong competitive abilities for soil nutrients and their capacity to degrade recalcitrant organic compounds such as lignin and cellulose (Arnstadt et al., 2016; Liu et al., 2023), contributing to nutrient enrichment and fungal dominance. These results highlight the critical role of litter physicochemical properties in shaping fungal community composition, with chemical traits serving as important indicators for microbial colonization during decomposition (Lin et al., 2021).

Litter decomposition in arid deserts plays a vital role in attenuating solar radiation, intercepting precipitation, enhancing soil organic matter, and sustaining microbial diversity (Liu et al., 2023). This study demonstrates that bacterial α-diversity—assessed via Chao1, Observed_species, and OTU count—was significantly greater in vegetated areas than in non-vegetated areas by 438.90–622.92, 373.88–576.54, and 573–1422, respectively, across both burial depth treatments (**Figures 4**, **6**), aligning with patterns observed in arid and semi-arid ecosystems (Bachar et al., 2012; Hortal et al., 2013). This disparity stems from the formation of nutrient-enriched microsites around vegetation patches—the so-called “fertile island” effect—which enhances soil biological availability and promotes microbial biomass and diversity in otherwise oligotrophic soils (Bachar et al., 2012). In contrast, fungal OTU numbers and α-diversity showed a more restricted spatial response: they were significantly higher only in surface soils of vegetated areas, with no significant difference in deeper layers, consistent with findings from He et al. (2023). This vertical stratification likely reflects the heightened sensitivity of microbial communities, particularly fungi, to vertical environmental gradients compared to horizontal heterogeneity (Guan et al., 2022). Notably, burial depth did not significantly influence bacterial or fungal α-diversity, indicating that the vertical environmental variation within the experimental gradient was insufficient to induce community differentiation—potentially due to a short experimental duration or minimal physicochemical differences between soil layers (He et al., 2023). However, habitat type had a significant effect on bacterial Chao1, Observed_species, and Shannon indices, reinforcing the dominant role of vegetation cover in shaping bacterial community assembly. Overall, habitat type, rather than burial depth, is the primary determinant of soil microbial community structure, especially for bacterial diversity. Vegetation presence substantially increases bacterial diversity and richness and promotes fungal diversity in surface soils, but this facilitative effect diminishes with increasing soil depth.

### 4.3 Regulation of litter decomposition by soil microorganisms and litter quality

The complete decomposition of litter is closely linked to the structure and function of the microbial community (Li et al., 2022). This study revealed that sand burial in vegetated areas significantly increased the number and diversity of microorganisms, which corresponded with an accelerated rate of litter decomposition. This phenomenon may be attributed to the increased availability of moisture, temperature, and nutrient sources within these environments (Witoon et al., 2016). Conversely, sand burial in non-vegetated areas did not promote litter decomposition, and no significant correlations were observed between dominant microorganisms and litter mass loss. These results suggest that litter decomposition in bare sandy habitats is primarily driven by abiotic factors such as photodegradation and physical fragmentation (Fan et al., 2021), while microbial contributions to decomposition are relatively limited (Austin and Vivanco, 2006; Austin et al., 2016; Qu et al., 2021). It is noteworthy that the relationship between litter mass loss and soil microbial α-diversity was not statistically significant in this study (**Supplementary Figure S4**), contrasting with findings from several recent studies that have reported positive correlations under similar ecological contexts (Qu et al., 2021; He et al., 2023; Liu et al., 2023). We propose that the reduction in microbial α-diversity in this study may not have been sufficient to exert a detectable influence on decomposition rates. Alternatively, the slow pace of litter turnover in desert ecosystems may delay the emergence of microbial effects on decomposition dynamics (Cornwell et al., 2008).

SEM results indicate that the C/N ratio and lignin content of litter are critical determinants of decomposition dynamics, consistent with previous findings (Yang et al., 2019b; Fan et al., 2021). Correlation analysis revealed a significant decline in the litter C/N ratio alongside increased mass loss. However, this reduction in the C/N ratio was observed only at the 15 cm sand burial depth in vegetated areas, where decomposition rates were highest, suggesting that sand burial enhances decomposition in microenvironments with vegetation. A plausible explanation is that vegetation cover increases microbial abundance and α-diversity, accelerating microbial biomass turnover and nutrient cycling, thereby facilitating a chemical stoichiometric balance between microorganisms and litter, altering microbial community structure, and promoting decomposition (Hossain and Sugiyama, 2020). Additionally, sand burial moderates the effects of solar radiation on litter while increasing soil moisture and temperature (Qu et al., 2021), thereby enhancing microbial N utilization and further facilitating litter decomposition. We also observed increased levels of lignin, *Actinobacteria*, and *Ascomycota* under vegetation cover, which positively influence litter mass loss (**Figure 8A**). This indicates that vegetation creates a more stable microenvironment, enhancing microbial colonization and metabolic activity of key functional groups, thereby promoting sustained biotic decomposition (Arnstadt et al., 2016; Ullah et al., 2023). As labile compounds such as cellulose and hemicellulose are preferentially degraded, recalcitrant lignin becomes relatively enriched (Ye et al., 2022). Under these conditions, *Actinobacteria*—due to their broad distribution and capacity to produce diverse hydrolases and oxidases—emerge as key bacterial taxa in the transformation of complex organic matter (Mackelprang et al., 2011), while *Ascomycota* demonstrate the ability to utilize lignin-derived compounds and dominate in mid- to late-stage decomposition (Zheng et al., 2021). Thus, the coordinated dynamics among lignin enrichment, *Actinobacteria* proliferation, and *Ascomycota* dominance reflect a characteristic biologically driven pattern in vegetated areas: shifts in substrate composition promote the enrichment of specialized microbial communities that accelerate residual decomposition through a positive feedback mechanism—likely contributing to higher decomposition efficiency in these ecosystems. In contrast, bare sandy areas showed no significant enhancement in microbial activity or litter mass loss (**Figure 8B**), yet exhibited lower surface lignin residues and higher overall decomposition rates. This discrepancy is best explained by solar radiation-mediated photodegradation: in unvegetated environments lacking canopy cover, high-intensity ultraviolet and visible light directly break down recalcitrant components such as lignin (Day et al., 2019; Austin and Ballaré, 2024; Li et al., 2024). Although structural equation modeling enables integration of multilayer ecological factors and visualization of complex interactions, the current analysis remains cross-sectional due to the absence of time-series or experimental perturbation data. Future studies should employ longitudinal designs or repeated measurements to better resolve temporal dynamics and strengthen causal inference regarding microbial community development.

SEM results further revealed that litter decomposition in non-vegetated areas significantly contributed to the accumulation of soil TC/TN ratios. This effect may be attributed to the increased growth and metabolic activity of *Proteobacteria* during decomposition, which accelerates the transformation of organic C and subsequently influences the TC/TN ratio in soils (Craig et al., 2022). In contrast, litter decomposition in vegetated areas did not lead to a net accumulation of soil TC/TN. This discrepancy may result from the additional organic inputs provided by vegetation, which meet microbial N demands and promote N mineralization and release into the soil matrix (Liu et al., 2010, 2019). These findings are consistent with previous research indicating that microbial communities respond to environmental disturbances by adjusting their C, N, and P utilization strategies. Specifically, microorganisms may regulate nutrient cycling by modulating the activity of extracellular enzymes, altering community composition, or adapting their internal stoichiometry to optimize resource acquisition and maintain elemental balance (Allison et al., 2013). This study, relying on microbial samples collected at a single time point, is limited in its ability to capture temporal dynamics of microbial community succession and cannot establish the causal temporal relationship between litter decomposition and soil C and N accumulation. Future research integrating time-series sampling with stable isotope tracing and functional omics approaches—such as metatranscriptomics or metaproteomics—could provide critical insights into the dynamic patterns of microbial functional activity and their role in driving soil element cycling. Such integrative methodologies would substantially improve the accuracy of mechanistic interpretations and deepen our understanding of ecosystem-level processes.

This study demonstrates that in bare sandy areas, the accumulation of organic matter and the development of ecologically functional soil layers cannot be effectively achieved through natural sand burial alone when vegetation is absent. Therefore, successful ecological restoration must prioritize vegetation establishment, particularly through the introduction of drought-tolerant and nutrient-poor-adapted native species. Their litter inputs play a critical role in improving soil physicochemical properties and facilitating microbial colonization. For artificial sand fixation, a synergistic “engineering + biological” approach is recommended, integrating sand barrier systems with simultaneous vegetation planting to construct stable, functionally complementary composite protection systems that enhance the durability of stabilization efforts and accelerate ecological succession. In monitoring, the dynamics of litter decomposition and associated shifts in soil microbial community structure should serve as key indicators for assessing restoration outcomes. These processes directly reflect ecosystem-level energy flow and material cycling, offering high sensitivity, quantifiability, and suitability for long-term evaluation. Regular measurements of mass loss rates in standardized litter bags, N and P release patterns, and microbial abundance enable dynamic assessment of different restoration strategies. Overall, this study advances understanding of ecological processes in arid ecosystems and strengthens the practical applicability of research findings, supporting the transition of desertification control from experience-based practices to science-informed governance and providing a robust foundation for developing more precise, effective, and sustainable ecological conservation policies.

## 5 Conclusion

In vegetated areas, sand burial significantly enhances litter decomposition rates, with its influence intensifying over time. In contrast, the decomposition rate of litter in non-vegetated areas remains relatively unchanged. Furthermore, sand burial in vegetated areas markedly increases the release of C and N from litter, reduces the C/N ratio, and accelerates overall decomposition. SEM results indicate that *Actinobacteria* and *Ascomycota* play key roles in driving litter decomposition in vegetated areas by facilitating lignin degradation. This suggests that sand burial promotes decomposition by reducing the C/N ratios and enhancing microbial activity—specifically that of *Actinobacteria* and *Ascomycota*. Notably, surface litter decomposed more rapidly in non-vegetated areas than in vegetated areas, implying that abiotic factors may dominate litter decomposition processes in extremely arid environments. Compared with the litter C/N ratios and soil fungal contributions, photodegradation-induced lignin breakdown may be the principal mechanism accelerating mass loss of surface litter in non-vegetated zones. As vegetation coverage expands and shades the soil surface, the influence of photodegradation is likely to diminish, making the decomposition of buried litter increasingly important for nutrient cycling and ecosystem stability in desert regions. This study advances our understanding of litter decomposition dynamics and microbial responses to extreme microhabitat heterogeneity. It underscores the critical roles of photodegradation, litter chemical traits, and soil microbial communities in regulating decomposition processes in arid environments.

## Data Availability

The raw data supporting the conclusions of this article will be made available by the authors, without undue reservation.
