# Supplementary material for: Alterations in microhabitat can impact litter decomposition by modifying the litter C/N ratio and regulating soil microbial activity
Source: Front Plant Sci. 2026 Jan 20;16:1660144. doi: 10.3389/fpls.2025.1660144 (PMC12864463; doi:10.3389/fpls.2025.1660144)
Supplement: Supplementary file 1 [file DataSheet1.docx]

**Specific methodological details for β-diversity analysis**

To assess differences in microbial community composition among treatments, the Bray-Curtis dissimilarity matrix was calculated and visualized using non-metric multidimensional scaling (NMDS), providing an ordination representation of community distribution patterns across treatment groups. While NMDS offers a useful graphical summary, it does not provide statistical inference regarding compositional differences. Therefore, permutational multivariate analysis of variance (PERMANOVA) was conducted using the adonis2 function in the vegan package to formally test for significant differences in community structure. This approach partitions the dissimilarity matrix to quantify the proportion of variation explained by experimental factors—such as habitat type, soil depth, or sampling time—and produces pseudo-F statistics, degrees of freedom, and associated P-values based on permutation tests (typically 999 permutations), allowing rigorous assessment of group-level differences. In addition, to verify the assumption of homogeneity of multivariate dispersions—a prerequisite for valid interpretation of PERMANOVA results—we performed a betadisper analysis, also implemented in the vegan package. This method computes the Euclidean distance of each sample to its group centroid in multivariate space and uses permutation tests to compare average dispersion among groups, thereby detecting potential differences in within-group variability that could influence the interpretation of between-group differences.

**TABLE S1** Alpha diversity index of bacteria and fungi of different treatments.

| Microbial species | Treat | Time | Chao1 | Observed_species | Shannon | Simpson |
| --- | --- | --- | --- | --- | --- | --- |
|  | VS | 0 | 3494.95±188.5a | 3131.83±146.5a | 9.51±0.2a | 0.99±0.004a |
|  |  | 12 | 2048.27±121.5ab | 1777.3±112.0ab | 7.31±0.2b | 0.97±0.004b |
|  | VB | 0 | 2994.07±50.7ab | 2804.33±72.9ab | 9.56±0.2a | 0.99±0.006a |
| Bacterial |  | 12 | 1650.1±78.5ab | 1435.5±54.6ab | 6.9±0.1b | 0.97±0.001b |
|  | NS | 0 | 2772.94±116.4ab | 2472.97±70.9ab | 8.92±0.2a | 0.99±0.004a |
|  |  | 12 | 1650.11±45.3ab | 1435.47±52.4ab | 6.89±0.10b | 0.97±0.003b |
|  | NB | 0 | 3018.04±65.6ab | 2802.37±45.9ab | 9.61±0.1a | 0.99±0.005a |
|  |  | 12 | 1376.65±112.6b | 1194.83±119.1b | 6.84±0.3b | 0.97±0.01b |
|  | VS | 0 | 224.11±7.01a | 219.37±6.94a | 4.11±0.04bc | 0.86±0.01ab |
|  |  | 12 | 133.64±3.56b | 131.17±3.23b | 3.93±0.08bc | 0.87±0.01ab |
|  | VB | 0 | 133.627±9.69b | 128.40±9.41b | 3.76±0.33bc | 0.86±0.04ab |
| Fungal |  | 12 | 110.56±8.12b | 109.11±8.16b | 3.91±0.14bc | 0.88±0.01ab |
|  | NS | 0 | 198.39±4.845a | 193.17±5.441a | 4.57±0.050ab | 0.87±0.005ab |
|  |  | 12 | 118.97±9.746b | 116.60±9.16b | 3.72±0.004bc | 0.84±0.001ab |
|  | NB | 0 | 204.89±4.419a | 198.67±4.863a | 5.01±0.027a | 0.91±0.009b |
|  |  | 12 | 127.78±3.278b | 126.73±3.963b | 4.17±0.162bc | 0.88±0.018ab |

Note: The difference in the diversity index between values for bacterial (fungal) is indicated by different lowercase letters (mean ± SE, n = 3, *P* < 0.05). Treatments are described in **Figure 1**.

**TABLE S2** Results of the adonis2 and betadisper analyses (*P* < 0.05).

| Species | Test type | *df* | *F* | *P* | R^2^ |
| --- | --- | --- | --- | --- | --- |
| Bacterial | Adonis2 | 1 | 1.890 | **0.001** | 0.155 |
|  | Betadisper | 1 | 2.142 | **0.158** |  |
| Fungal | Adonis2 | 1 | 2.654 | **0.012** | 0.102 |
|  | Betadisper | 1 | 1.38 | **0.256** |  |


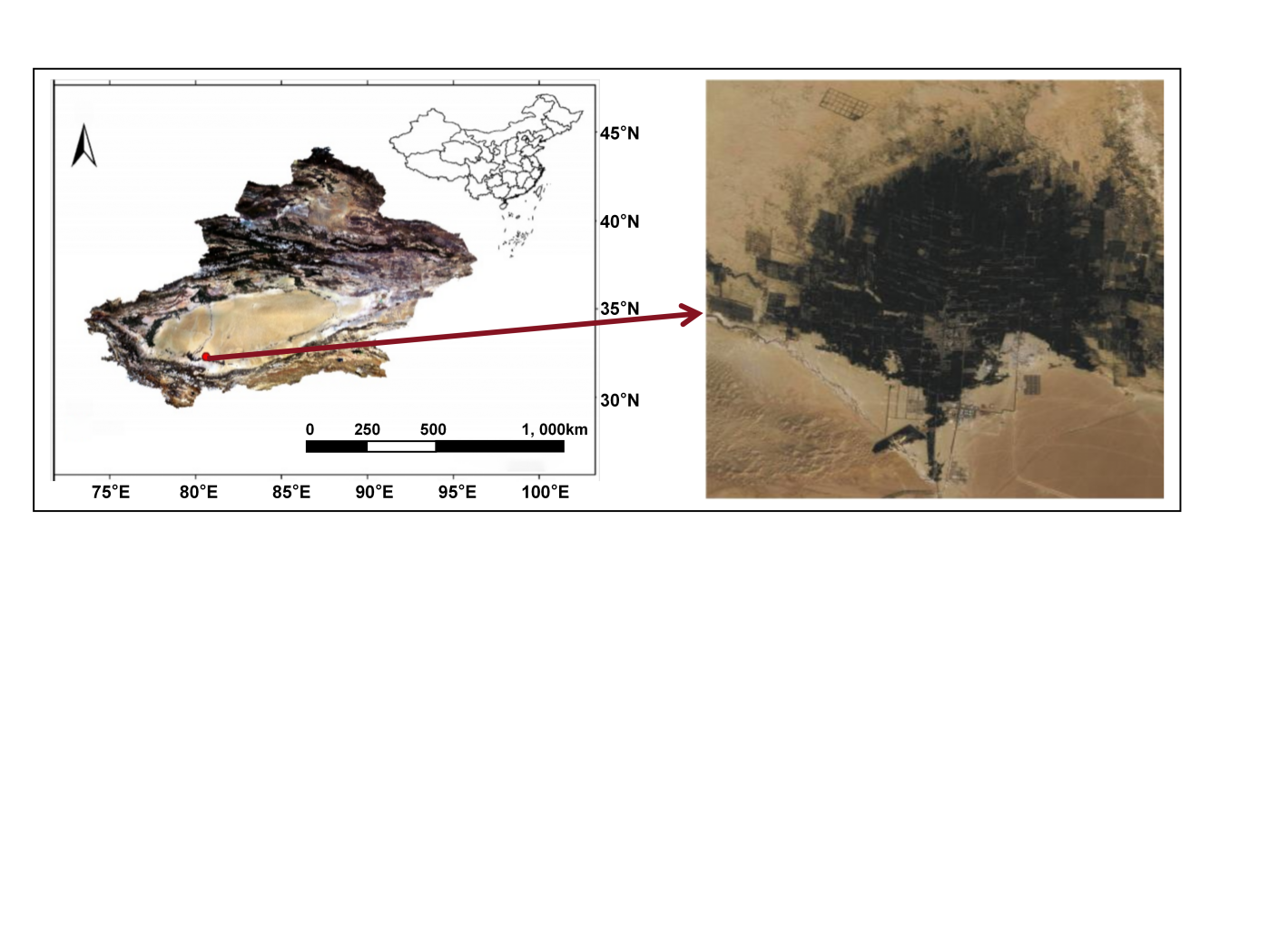


**FIGURE 1**

Illustrations depicting the experimental study sites.


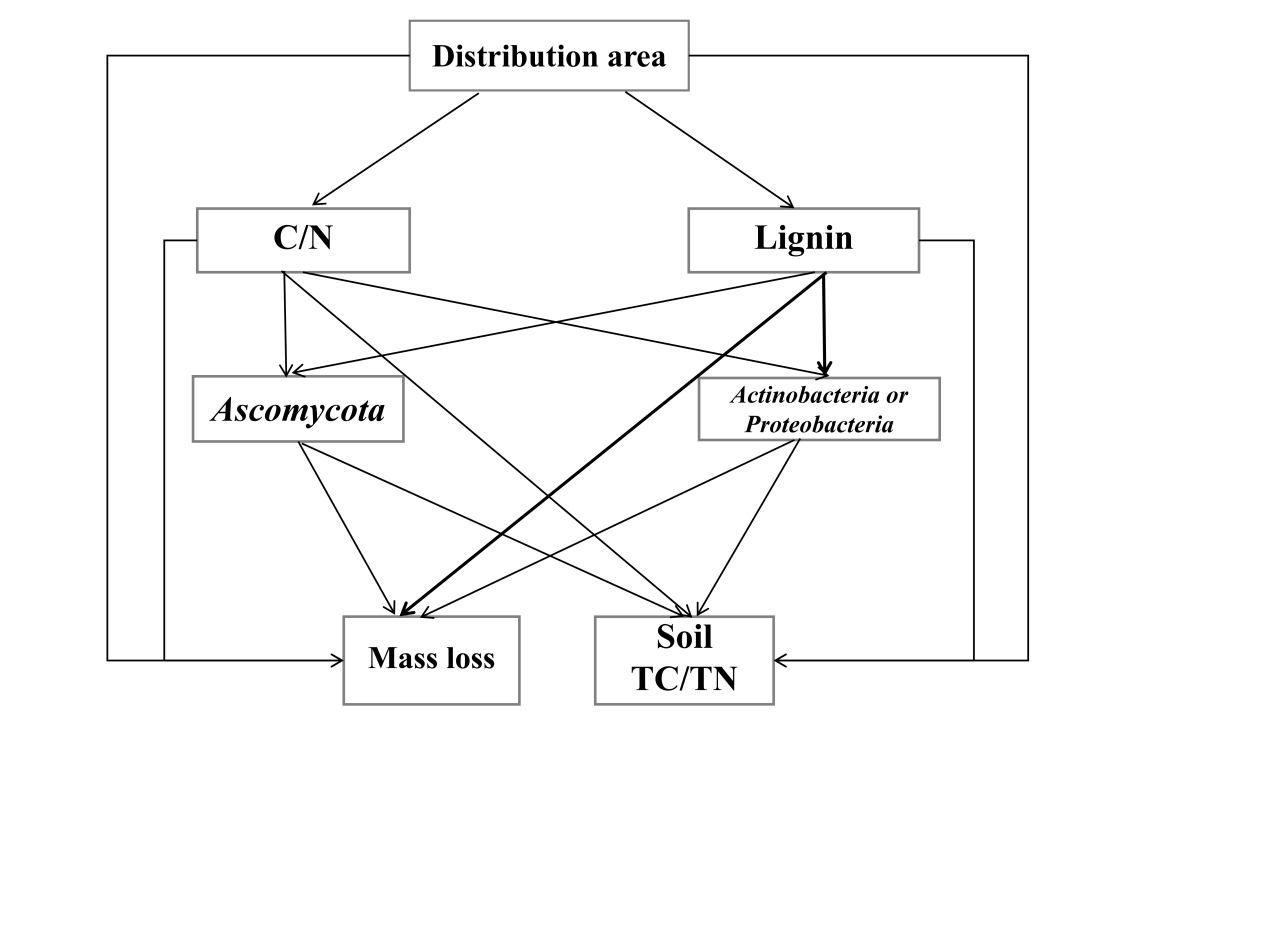


**FIGURE 2**

A priori model for structural equation modeling of litter decomposition under different habitats.


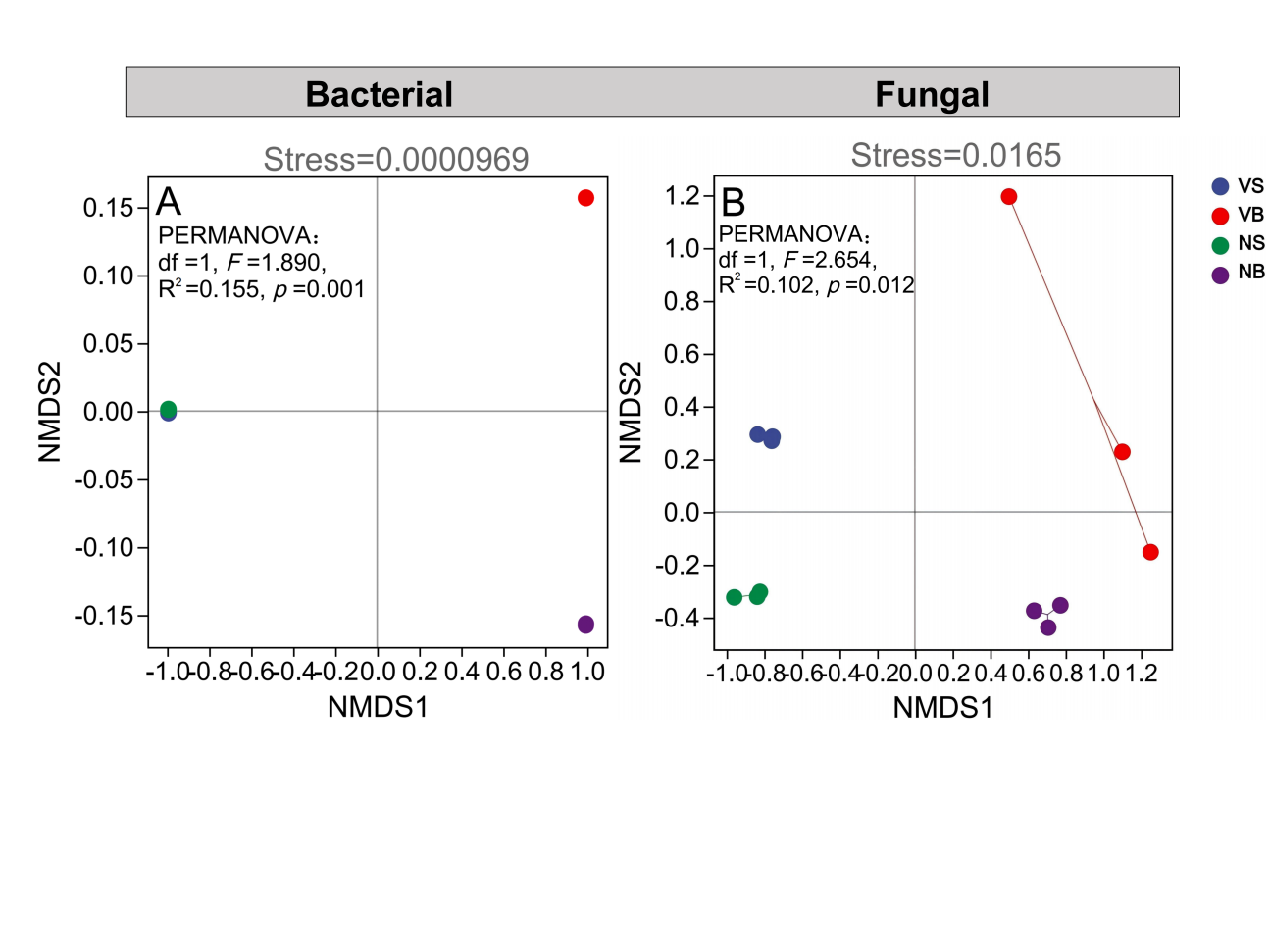


**FIGURE 3**

Non-Metric Dimensional Scaling (NMDS) plot of soil bacterial (A) and fungal (B) community structures based on the Bray-Curtis distance algorithm. Treatments are described in **Figure 1**.


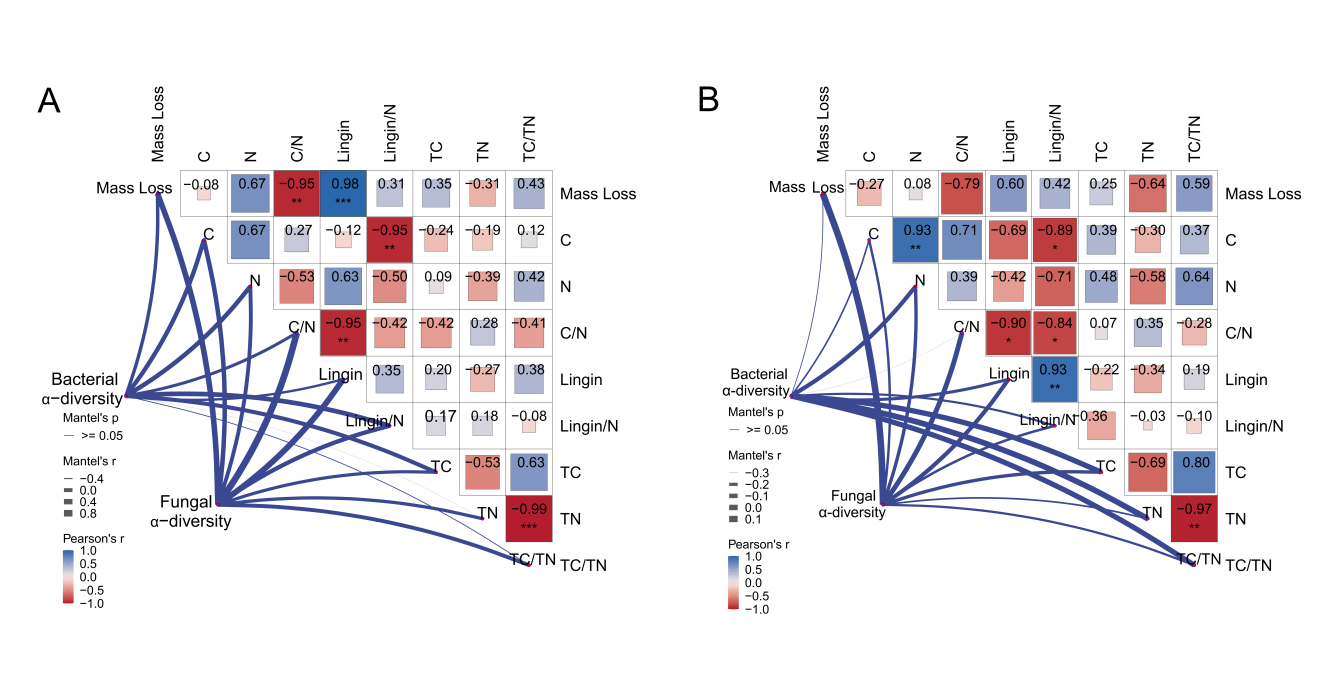


**FIGURE 4**

The correlation of litter mass loss, chemical composition and soil physicochemical properties, and the relationship between these environmental parameters and soil bacterial and fungal α-diversity of vegetation area (A) and no-vegetation area (B). Pairwise comparisons of environmental parameters are shown with a color gradient representing Pearson's correlation coefficient. The width of the edge corresponds to the Mantel’s r value, and the edge color indicates statistical significance.


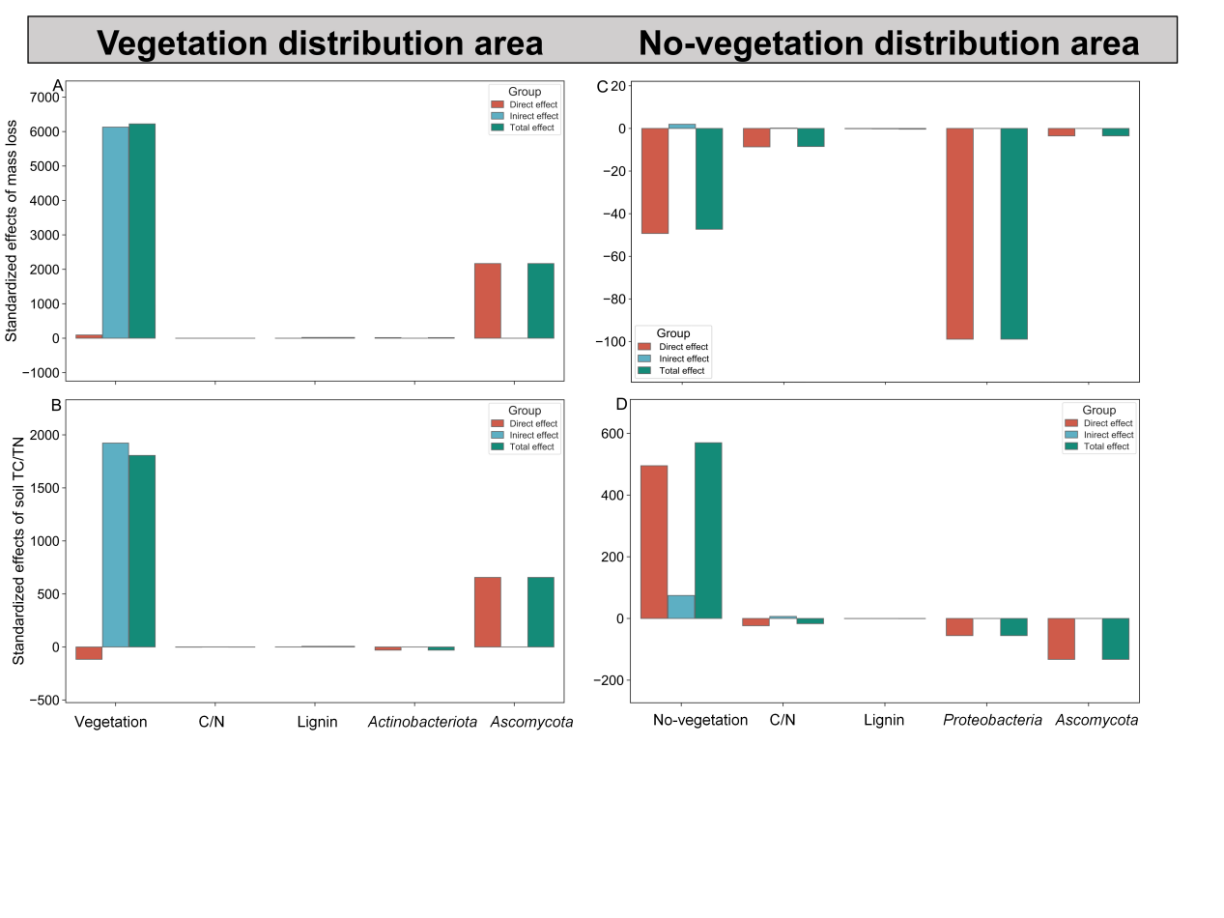


**FIGURE 5**

The standardized effects of litter properties and dominant soil microbial communities on litter mass loss and soil TC/TN ratio.


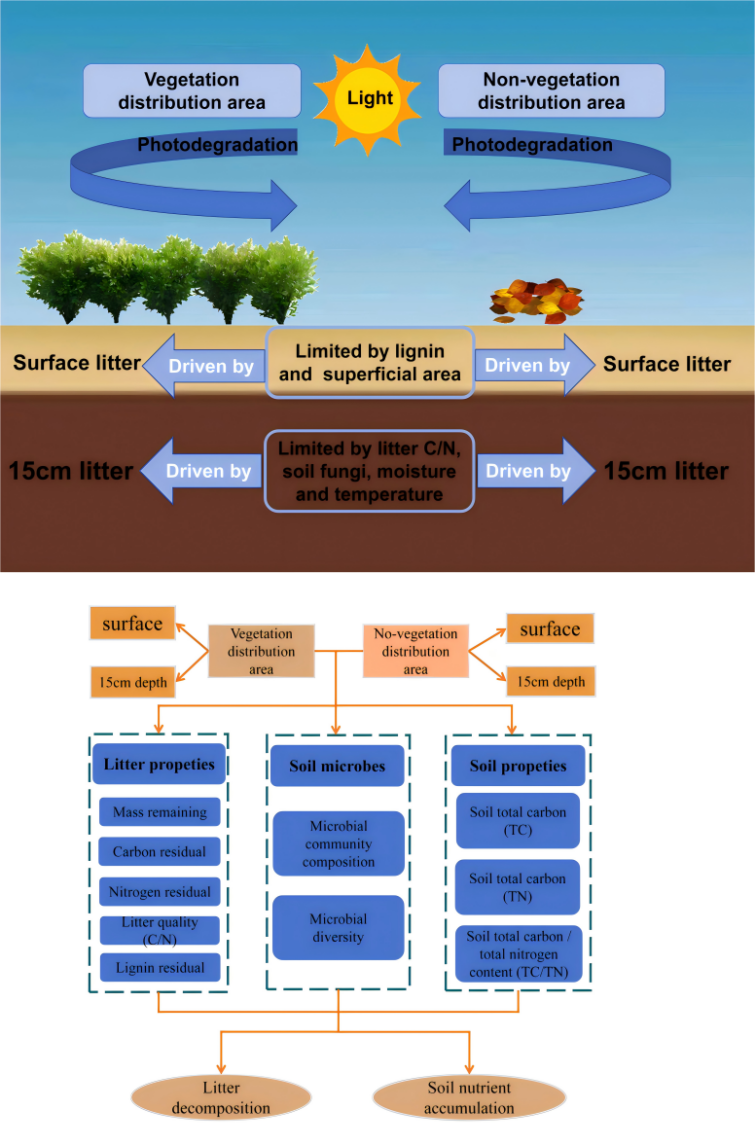


**FIGURE 6**

Graphical summaries in different habitats.
